# Potential Novel Tissue Biomarkers in Salivary Glands of Patients with Sjogren’s Syndrome

**DOI:** 10.3390/jcm14072390

**Published:** 2025-03-31

**Authors:** Canan Sadullahoglu, Neslihan Yaprak, Veli Yazısız, İrem Hicran Ozbudak

**Affiliations:** 1 Department of Pathology, Antalya Training and Research Hospital, 07100 Antalya, Turkey; canan-rana@hotmail.com; 2 Department of Otorhinolaryngology, Antalya City Hospital, 07080 Antalya, Turkey; 3 Department of Rheumatology, Faculty of Medicine, Akdeniz University, 07070 Antalya, Turkey; vyazisiz@akdeniz.edu.tr; 4 Department of Pathology, Faculty of Medicine, Akdeniz University, 07070 Antalya, Turkey; iremhicrang@hotmail.com

**Keywords:** primary Sjogren’s syndrome, SSA/Ro, SSB/La, BAFF, MMP-9

## Abstract

**Background/Objectives:** Primary Sjogren’s syndrome (pSS) is an autoimmune disease caused by chronic inflammation of the exocrine glands. We aimed to determine the immunohistochemical parameters that may help in the diagnosis of pSS and to determine histopathological markers for the early diagnosis of patients. **Methods:** Hematoxylin–eosin-stained preparations of salivary gland tissue samples of a control group consisting of 9 healthy patients and 12 patients diagnosed with non-specific chronic sialadenitis (NSCS) and a study group consisting of 39 patients diagnosed with pSS were evaluated. SSA/Ro (TRIM21), SSB/la, BAFF, enolase (ENO1), and MMP-9 antibodies were applied to the samples to assess the presence of staining in the ductal, acini, and inflammation regions. **Results:** In our study, mild staining with the SSA/Ro (TRIM21) antibody was observed in the ductal areas of all pSS cases, while no staining was detected in the healthy and NSCS cases (*p* < 0.01). Additionally, stronger staining was identified in the ductal and inflammatory areas of the pSS cases with BAFF compared to the control group. The staining for the ENO1 antibody was higher and more intense in the NSCS and pSS cases compared to those with normal salivary glands, and this difference was found to be statistically significant (*p* < 0.01). While mild staining was detected in the ductal areas with MMP-9 antibody in most of the NSCS and pSS cases, it was detected in 11% of the healthy cases. **Conclusions**: Our study suggests that SSA/Ro (TRIM21), ENO1, MMP9, and BAFF can be used to confirm the diagnosis in cases of suspicion.

## 1. Introduction

Primary Sjogren’s syndrome (pSS) is a chronic inflammatory disease of the exocrine glands without any other autoimmune disease. It is characterized by the presence of autoantibodies against Ro/SSA and La/SSB, which are developed against ribonucleoprotein (RNP) particles of exocrine tissues (especially the salivary and lachrymal glands), and mononuclear cell infiltration [1,2]. A multidisciplinary approach, including rheumatology and ophthalmology, leads to the diagnosis of SS based on clinical, laboratory, and histopathological findings [1,2,3,4]. The serological detection of anti-SSA/Ro autoantibodies, which occur in 67% of SS cases, and anti-SSB/La autoantibodies, which are detected in 49% of cases, plays an essential role in diagnosis and prognosis [1]. There are cases in the literature that are anti-SSA/Ro- and anti-SSB/La-negative, along with those that are anti-nuclear antibody (ANA)-positive. This group has been referred to as “seronegative SS”. Therefore, studies are being conducted to find new biomarkers in the serum that contribute to diagnosis and early detection. It has been reported that serum BAFF levels increase in many autoimmune diseases such as SLE, pSS, RA, and immune thrombocytopenia (ITP) [5]. Overexpression of α-enolase has been detected in chronic autoimmune diseases such as RA, systemic sclerosis, and primary nephropathies. Autoantibodies against α-enolase are detected very early in the sera of patients with RA and have been reported to have potential diagnostic and prognostic value [6]. Wei et al. reported that the overexpression of α-enolase and high antibody levels in the serum might be associated with salivary gland hypofunction and inflammatory immune responses. Therefore, α-enolase expression can be used as a biomarker for pSS [7]. SS patients are reported to have significantly higher levels of MMP-9 in their saliva and labial salivary glands compared to healthy controls [8,9]. Considering the pathogenesis of SS, MMP-9 activation causes significant changes in salivary gland parenchyma, extracellular matrix structure, and functions by destroying many extracellular matrix components, including type IV–V collagen and elastin [8].

Additionally, minor salivary gland biopsy, which is minimally invasive, is an important test helpful in diagnosing the disease [4]. Its sensitivity has been reported to be 63.9–85.7%, and its specificity 61.2–100%, for minor salivary gland biopsy [10]**.** Although focal lymphocytic sialoadenitis, one histopathological finding, is a necessary criterion for diagnosis, there are differences between pathologists in the evaluation. In addition, studies have reported that, in approximately half of the cases with focal lymphocyte sialoadenitis, no antibodies against Ro and La antigens were detected serologically. This shows that most patients cannot be clinically diagnosed without a salivary gland biopsy [4]. In addition, in biopsy specimens from patients with pSS, diffuse mild-to-moderate acinar atrophy, interstitial fibrosis, ductal dilatation, non-specific chronic sialoadenitis, sclerosing chronic sialoadenitis, granulomatous inflammation, and lymphoid follicular cells are also observed [10].

SS is difficult to diagnose early, but late diagnosis is more likely to result in severe systemic complications and lymphoproliferative disease. Therefore, different biomarkers are recommended for diagnosing and treating SS and its subtypes. In this regard, it is anticipated that identifying specific markers will assist in developing more personalized treatments and better outcomes [1].

This study aimed to evaluate the tissue expression of serological markers used in daily practice and investigate their diagnostic value.

## 2. Materials and Methods

### 2.1. Patient Selection

Salivary gland biopsies with pSS samples submitted to Akdeniz University Faculty of Medicine, Department of Pathology, between 1 January 2006 and 31 December 2018, were scanned from the archive. The inclusion criteria for the study were cases with paraffin blocks and preparations suitable for diagnosis and examination, and the exclusion criteria were cases without paraffin blocks in the archive and not enough tissue in the paraffin blocks. Among these cases, 39 pSS cases with clinical follow-up were selected as the study group. As the control group, 12 non-specific chronic sialoadenitis and 9 healthy salivary gland cases were chosen. All the cases were evaluated using the Chisholm and Mason ACR/EULAR 2016 Classification criteria. In addition, a hospital automation system was used to obtain demographic and clinical information about the patients.

pSS samples and salivary gland biopsies sent to Akdeniz University Faculty of Medicine Pathology Department between 1 January 2006–31 December 2018 were scanned from the archive. The histological and immunohistochemical evaluation was conducted by Canan Sadullahoglu. The tissue area (in micro- and square millimeters) was measured microscopically using an image processing-based diagnosis and analysis system (argentite/camera 5) in patients diagnosed with Sjogren’s syndrome in preparations stained with hematoxylin–eosin. The number of focal aggregates containing at least 50 lymphocytes in an area of 4 mm^2^ in the periductal or perivascular region and the focus score were calculated to identify focal lymphocytic sialadenitis, the histopathological diagnosis supporting the disease in biopsy specimens. Grading was conducted following the Chisholm Mason classification (Grade 0: normal; Grade 1: mild; Grade 2: moderate mononuclear cell infiltration; Grade 3: the presence of one focus; Grade 4: the presence of multiple foci).

### 2.2. Immunohistochemical Application and Evaluation

For IHC evaluation, 4-micron sections were taken on a positively charged slide for each antibody from the paraffin blocks of the patients included in the study. The sections were kept at 50 °C overnight. Different dilutions for each antibody and positive controls were used for IHC staining (Table 1). Anti-SSA/Ro, anti-SSB/La, anti-enolase, anti-BAFF, and MMP-9 antibodies were applied on the sections taken using the standard procedure in a fully automatic Daco staining machine (Doca, FL, USA) and analyzed under a light microscope.

The expression of antibodies was evaluated in ductal and acinar areas for all cases, while the inflammatory area was assessed in 12 NSCS and 39 pSS cases. The staining intensity was graded using three categories: mild, medium, and strong. A lack of staining with the BAFF, TRIM21, MMP9, ENO1, and SS-B antibodies was considered negative staining. Linear membranous and cytoplasmic staining were accepted as positive staining, with BAFF, TRIM21 (SS-A/Ro), MMP9, and ENO1 (α-enolase) as complete or partial. The nuclear expression of SS-B was evaluated as positive staining.

### 2.3. Statistical Analysis

The Statistical Package for the Social Sciences (SPSS), version 17.0, program was used for statistical analysis. The study data were evaluated using descriptive statistical methods (mean, standard deviation, frequency, percentage, minimum, and maximum). In addition, the Pearson chi-square test and Fisher’s exact test were used to compare qualitative data. Statistical significance was defined as *p* < 0.05.

## 3. Results

Of the 60 cases included in the study, 90% (n = 54) were female and 10% (n = 6) were male. The subjects ranged in age from 24 to 72; the mean age was 48.8 (Std ± 11.8).

**Figure 1:**
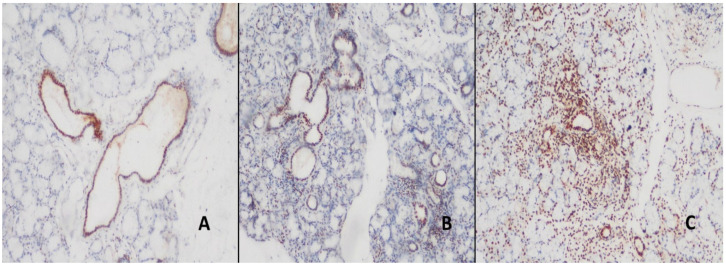
(**A**) Staining with SS-B antibody in the normal salivary gland and nuclear staining in the ductus, acini and inflammation areas. (DAB, ×200). (**B**) Nuclear staining in the ductus, acini, and inflammation areas in the patient with NSCS (DAB, ×100). (**C**) In patients with pSS, a relatively more widespread nuclear staining was observed in the ductal, acinar, and inflammatory areas (DAB, ×100).

Nuclear expression was detected in all the ducts and inflamed areas in the control and study groups by means of the SS-B antibody. There were no statistically significant differences in the differentiation between control and study groups. However, while focal SS-B expression was observed in the acini of the control group, SS-B expression was relatively high in the study group (Figure 1).

**Figure 2:**
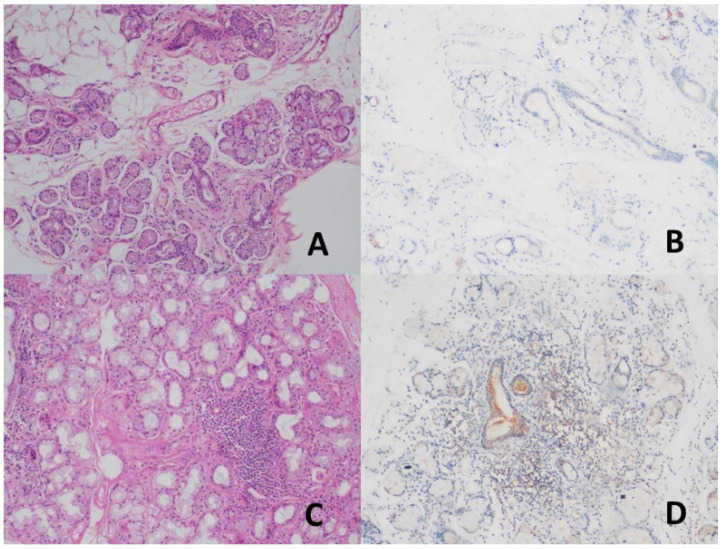
(**A**,**B**) No staining was observed in the duct areas in the normal salivary gland (H&E, ×200) (DAB, ×200). (**C**,**D**) Mild staining was detected in the duct and inflammation areas in patients with primary Sjogren’s syndrome (H&E, ×200) (DAB, ×200).

While no staining was observed in the duct areas in the control group with TRIM21 (SS-A/Ro52) antibody, mild staining was detected in the ducts in the study group diagnosed with pSS (Figure 2). TRIM21 expression significantly differed between the study and control groups (*p* < 0.01) (Table 2). Mild expression was found in 51.3% of the patients with pSS, whereas no expression was found in patients with NSCS in the control group (Table 3). A statistically significant difference was found between the control and pSS groups in terms of TRIM21 antibody expression (*p* < 0.01). The acini areas showed mild TRIM21 staining in all cases.

**Figure 3:**
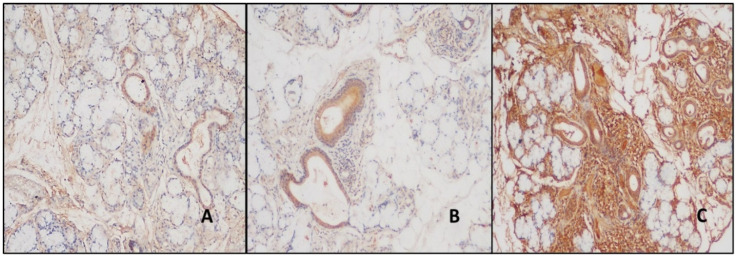
(**A**) Staining in duct areas with BAFF antibody in normal salivary gland (DAB, ×200). (**B**,**C**) The ducts and areas of inflammation were stained more in patients with pSS than in patients with NSCS (DAB, ×200).

BAFF expression was observed in the ducts in all cases. There was mild staining in the ducts of 42.8% of the normal and 33.4% of the NSCS cases in the BAFF control group, but no intense staining was detected. Strong staining was detected in 48.8% of the pSS cases (Table 2). This intense staining was statistically significantly different between the study and control groups (*p* < 0.01). The expression of BAFF in areas of inflammation was observed in 75% of the NSCS patients and in all the pSS patients. It was not statistically significant. However, the inflamed areas of the NSCS patients had mild staining in 66.7% of cases, whereas patients with pSS had moderate and strong staining in more significant percentages (Figure 3). The BAFF expression rates are detailed in Table 3. All the acini areas remained stain-free.

**Figure 4:**
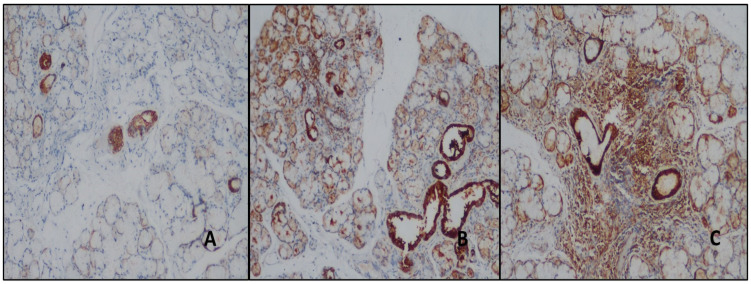
(**A**) Moderate staining in duct areas with ENO1 antibody in normal salivary gland (DAB, ×100). (**B**,**C**) In cases with NSC and pSS, stronger staining was detected in the duct areas, and in the cases with pSS, stronger staining was detected in the areas of inflammation than in the patients with NSCS (DAB, ×100).

Moderate and strong staining was detected in the ducts with ENO1 in both the study and control groups. Moderate staining was detected in all normal salivary gland biopsies duct areas, and strong expression was observed in all the NSCS and study group cases (Figure 4). The difference in ENO1-antibody expression was statistically significant in the differential diagnosis of normal salivary gland and NSCS and pSS (*p* < 0.01) (Table 2). In total, 66.7% of the patients with NSCS were ENO1-antibody-negative in the inflamed areas; however, mild staining was detected in 33.3% of them. Meanwhile, 20.5% of the patients with pSS showed mild, 46.2% moderate, and 33.3% intense staining (Figure 4) (Table 3). The difference was statistically significant (*p* < 0.01).

**Figure 5:**
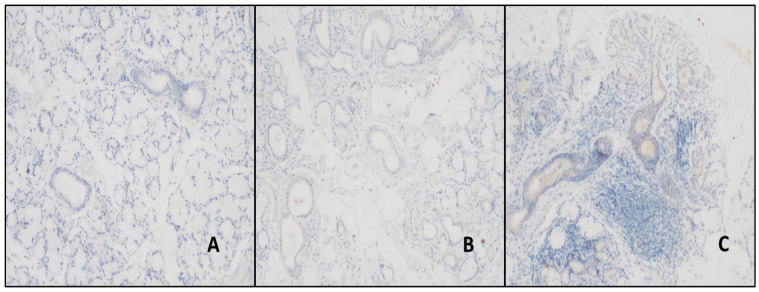
(**A**–**C**) While no MMP9 antibody was detected in normal salivary gland ducts, similar staining was found in these areas in patients with NSCS and PSS (DAB, ×100).

A total of 52.4% of the control group had MMP-9 antibody in the duct areas; 4.8% had antibodies in the normal biopsy subgroup; 47.6% had antibodies in the NSCS subgroup. Mild staining was detected in 84.6% of the study group (Figure 5) (Table 2). The study and control groups did not differ statistically significantly (*p* = 0.07) (Table 2). Acini and inflamed areas were not stained with MMP-9 in any case. 

## 4. Discussion

Primary Sjogren’s syndrome (pSS) is an autoimmune disease that is difficult to clinically diagnose [11]; to diagnose pSS, serum SS-A/Ro and SSB/La antibodies are assessed clinically [12]. Serological tests are positive in approximately half of all pSS cases [1]. Serological data are diagnostic when combined with clinical findings, but their sensitivity is low. Consequently, it is necessary to find different biomarkers and apply different techniques. Considering the pathogenesis of pSS, it is known that tissue damage occurs with autoantibodies before clinical findings appear [1,13]. The literature on IHC in salivary gland biopsies is limited.

While serum anti-SSB/La positivity is highly specific, its sensitivity (40%) in diagnosing pSS is relatively low compared to that for autoimmune disorders like SLE [14]. Currently, no IHC study has been conducted on salivary gland tissue in patients with SS. Our research detected nuclear staining in all the ducts, acini, and inflammation areas in the control and study groups with SSB/La antibodies. Staining was noted in the nuclei of the acini, although it was more prevalent in the group with pSS than in the patients with NSCS. When the NSCS and pSS groups were compared with standard salivary gland samples, expression was relatively weak in normal salivary gland acini. Based on these findings, artificial intelligence-based algorithms may be able to standardize evaluations and find meaningful breakpoints.

To diagnose pSS, SS-A/Ro (TRIM21) are routinely examined in the serum by the clinic. In the study by Aqrawi et al., Ro52 (SS-A)/TRIM21 expression in the salivary gland was evaluated; it was found that the ductal epithelium showed stronger expression in the patient group than in the control group. Additionally, strong expression was observed in the focal infiltration areas in all cases of pSS [15]. Another study found high levels of Ro52/TRIM21 expression in acinar and ductal areas in the control and study groups [16]. Similarly to the results in the literature, mild staining was detected in the study group, while no staining was observed in the control group. In addition, there was no expression in patients with NSCS containing an area of inflammation in the control group, while 51% of patients with pSS had mild expression. Unlike in previous studies, we observed mild acinar staining in all cases.

The BAFF levels in the serum, saliva, and lacrimal salivary gland have been found to be elevated in autoimmune diseases such as SLE, pSS, and RA [17,18]. Phase II studies using BAFF and BAFF receptor inhibitors reported improvements in symptoms such as dryness, pain, and fatigue in patients with Sjögren’s syndrome [19,20]. In a study conducted by Carrillo Ballesteros et al., BAFF expression exhibited a homogeneous distribution across the minor salivary glands of 29 patients, which included 4 NSCS cases, 2 NSCS-pSS cases, 16 pSS cases without germinal centers (GCs), and 7 pSS cases with germinal centers. No significant differences were observed between pSS patients with and without germinal centers. However, image analysis demonstrated distinct differences in the percentage of BAFF staining positivity between pSS-GC(+) patients and NSCS patients (pSS-GC(+) = 44.72% vs. NSCS = 6.43%) [18]. Similarly, our study found that pSS patients exhibited pronounced BAFF expression in ductal and inflammatory areas, while mild-to-moderate expression was noted in the NSCS cases.

A limited study reported that α-enolase (ENO1) was overexpressed in the minor salivary glands of pSS cases [7,21]. In a study by Wei et al., increased expression of ENO1 according to IHC in ductal and acinar areas was found in five pSS cases compared to four healthy controls. In addition, no expression in regions of inflammation was reported in five pSS cases [7]. Similarly, our study found that cases with pSS and NSCS showed more robust expression in the duct areas than normal salivary gland cases, and the difference was statistically significant (*p* < 0.01). While Wei et al. did not detect expression in the inflammatory areas of patients with pSS, our study identified varying levels of expression in all the pSS cases. Additionally, mild expression was observed in 33.3% of the NSCS cases. In some of the studies in the literature, MMP-9 was detected in acinar and ductal cells in pSS cases [22,23]. Recent studies conducted on animal models of Sjögren’s syndrome have shown that the increased expression of MMPs by lymphocytic infiltrates and epithelial cells in the lacrimal and salivary glands inhibits tissue repair and tear secretion. It has been demonstrated that the use of specific inhibitors of MMP-2/9 significantly improves the structure and secretion function of the lacrimal gland by inhibiting their activity [23]. In a study investigating the expression of matrix metalloproteinases in labial salivary glands, the expression of MMP-9 showed more pronounced and intense staining in the ducts and acini of 19 cases of pSS compared to 7 control cases that exhibited lymphocytic infiltration [8]. In a study investigating the MMP/TIMP balance in labial salivary glands, comprising 10 healthy control cases and 16 cases of pSS, staining was observed in the acinar and ductal areas of all cases. The expression of MMP-9 in the acini and ductal areas was increased in the pSS cases compared to a healthy control case [24]. In our study, mild expression was observed in the ducts in 84.6% of the patients with pSS and 83.3% (10/12) of those with NSCS. However, MMP-9 expression was found in the ducts of 11% (1/9) of the normal control group. No expression was observed in acini, and inflamed areas were not stained in any of the groups with MMP-9.

Studies have reported that histopathological findings such as focal lymphocytic sialoadenitis, ductal dilatation, adipose tissue infiltration, and fibrosis can be seen as part of the aging process in healthy individuals [25]. In addition, the observation of other histo-morphological findings (such as NSCS and sclerosing sialoadenitis) and focal chronic sialoadenitis in salivary gland biopsies used to define the disease may cause difficulties in the interpretation of biopsies [11]. Sialendoscopy is used for treatment of chronic obstructive disorders (such as strictures, mucus plugs, and sialoliths) [26,27,28]. The procedure is not especially diagnostic [29]. A limited number of studies have shown that sialendoscopy reduces the symptoms of Sjögren’s syndrome (SS) and improves salivary function [26,27,28]. In a study where the classification of Wharton’s duct papillae was based on the appearance of the papilla, dilation probes, and sialendoscopic procedures, all three patients with Sjögren’s syndrome were categorized as having normal papillae with a prominent orifice (Type B). In this study, 49.5% of sialolithiasis patients were classified as Type B, while all cases of radioiodine-induced sialadenitis and intraductal mass fell into this category [29].

The most important limitation of our retrospective study is that some of the antibodies whose expression was examined in the salivary gland biopsies were not used for diagnostic purposes in routine practice, and therefore, these antibodies were not compared with clinical parameters. Additionally, our patients could not be compared with the current findings because a sialendoscopic evaluation was not performed.

## 5. Conclusions

Although the detection of the expression of each antibody at varying rates in different regions of the salivary gland biopsy limits the routine use of these markers, evaluating the expression levels of TRIM21 (Ro52), ENO1 (α-enolase), MMP9, and BAFF in patients with pPSS may be helpful for supporting the diagnosis. However, more studies with larger series are needed for these markers. We believe that studies comparing serum antibody levels and immunohistochemical tissue markers may contribute to the early identification of the disease and assist in diagnosis in cases where diagnostic challenges arise. In addition, it may identify potential therapeutic targets for inhibitors to be developed against these antibodies.

## Figures and Tables

**Table 1:** The primary antibodies used, the dilution ratios, and the positive control tissues.

Primary Antibody	Company	Dilution Rate	Positive Control
Anti-BAFF antibodies [ab217329], RabPAb	abcam (Cambridge, UK)	1/150	Tonsil
Anti TRIM21 (SS-A/Ro) antibodies [ab191690], RabPAb	abcam	1/100	Tonsil
Anti MMP9 antibodies [56-2A4, ab 58803], MauseMAb	abcam	1/100	Tonsil
Anti ENO1 antibodies [EPR19758, ab 227978], RabMAb	abcam	1/200	Pancreas
Anti SS-B antibodies [EPR6570, ab 124932], RabMAb	abcam	1/250	Colon

**Table 2:** Staining of duct areas with TRIM21, BAFF, ENO1, and MMP-9 antibodies in the control and study groups.

Antibody	Control Group (n = 21)								Study Group pSS (n = 39)				*p* Value
	Normal (n = 9)				NSCS (n = 12)								
	N	P			N	P			N	P			
		M	MD	S		M	MD	S		M	MD	S	
TRIM21	9 42.8%				12 57.2%					39 100%			*p* < 0.01
BAFF		9 42.8%				7 33.4%	5 23.8%			5 12.8%	15 38.4%	19 48.8%	*p* < 0.01
ENO1			9 42.8%					12 57.2%				39 100%	*p* < 0.01
MMP-9	8 38.1%	1 4.8%			2 9.5%	10 47.6%			6 15.4%	33 84.6%			*p* = 0.07

NSCS: non-specific chronic sialoadenitis; pSS: primary Sjogren’s syndrome; N: negative; P: positive; M: mild; MD: moderate; S: strong.

**Table 3:** Staining with TRIM21, BAFF, and ENO1 antibodies in areas of inflammation in the control group (in patients with non-specific chronic sialoadenitis) and primary Sjogren’s syndrome.

Antibody	NSCS (n = 12)				pSS (n = 39)			
	N	P			N	P		
		M	MD	S		M	MD	S
TRIM21	12 100%				19 48.7%	20 51.3%		
BAFF	3 25%	8 66.7%	1 8.3%			4 10.3%	16 41%	19 48.7%
ENO1	8 66.7%	4 33.3%				8 20.5%	18 46.2%	13 33.3%

NSCS: non-specific chronic sialoadenitis; pSS: primary Sjogren’s syndrome; N: negative; P: positive; M: mild; MD: moderate; S: strong.

## Data Availability

Dataset available on request from the authors.
